# Oxidized Lipoproteins Suppress Nitric Oxide Synthase in Macrophages: Study of Glucocorticoid Receptor Involvement

**DOI:** 10.1155/S096293519400044X

**Published:** 1994

**Authors:** K. E. Matthys, P. G. Jorens, B. Marescau, M. Rosseneu, H. Bult, A. G. Herman

**Affiliations:** 1 Division of Pharmacology University of Antwerp (UIA) Wilrijk Belgium; 2 Division of Neurochemistry University of Antwerp (UIA) Wilrijk Belgium; 3 Department of Clinical Chemistry A.Z. St.-Jan Brugge Belgium

## Abstract

Activated cholesterol-laden macrophages in atherosclerotic lesions are believed to influence the progression of this disease. The induction of nitric oxide synthase (iNOS) activity was investigated in control and cholesterol-laden J774 macrophages, obtained by pre-incubation with oxidized or acetylated low density lipoproteins (oxLDL, acLDL). Loading with oxLDL caused a small induction of NOS activity in unstimulated cells, as indicated by nitrite and citrulline accumulation in the supernatant. However, it suppressed the iNOS activity resulting from stimulation of the cells with lipopolysaccharide with or without interferon-γ. AcLDL had no inhibitory effect, indicating that cholesterol accumulation as such was not responsible. Since the induction of NOS in macrophages is inhibited by glucocorticoids, the possibility that a glucocorticoid-like factor, formed during oxidation of LDL, may cause the inhibition, was investigated. However, addition of the glucocorticoid receptor antagonist mifepristone did not prevent the oxLDL-dependent NOS inhibition, indicating that the glucocorticoid receptor is not involved in the suppressive effect of oxLDL.


Mediators of Inflammation 3, 323–327 (1994)

### Oxidized lipoproteins suppress nitric oxide synthase in macrophages: study of glucocorticoid receptor involvement

K. E. Matthys,[1,CA] P. G. Jorens,[1] B. Marescau,[2] M. Rosseneu,[3] H. Bult[1] and A. G. Herman[1]

[1]Division of Pharmacology and [2]Neurochemistry, University of Antwerp (UIA), Wilrijk, Belgium, and [3]Department of Clinical Chemistry, A.Z. St.-Jan, Brugge, Belgium

[CA] Corresponding Author

**Activated cholesterol-laden macrophages in atherosclerotic lesions are believed to influence the progression of this disease. The induction of nitric oxide synthase (iNOS) activity was investigated in control and cholesterol-laden J774 macrophages, obtained by pre-incubation with oxidized or acetylated low density lipoproteins (oxLDL, acLDL). Loading with oxLDL caused a small induction of NOS activity in unstimulated cells, as indicated by nitrite and citrulline accumulation in the supernatant. However, it suppressed the iNOS activity resulting from stimulation of the cells with lipopolysaccharide with or without interferon-γ. AcLDL had no inhibitory effect, indicating that cholesterol accumulation as such was not responsible. Since the induction of NOS in macrophages is inhibited by glucocorticoids, the possibility that a glucocorticoid-like factor, formed during oxidation of LDL, may cause the inhibition, was investigated. However, addition of the glucocorticoid receptor antagonist mifepristone did not prevent the oxLDL-dependent NOS inhibition, indicating that the glucocorticoid receptor is not involved in the suppressive effect of oxLDL.**

**Key words:** Foam cell, Lipoprotein, Macrophage, Mifepristone, Nitric oxide synthase

### Introduction

The early stage of atherosclerosis is characterized by the infiltration of monocytes in the subendothelial space.[1] By the unregulated uptake of oxidized low density lipoproteins (oxLDL) through the scavenger receptor,[2] these mononuclear cells then accumulate large amounts of lipids and acquire a so-called 'foamy' appearance.

Macrophages in general produce numerous mediators which play a role in host defence and may therefore also affect the progression of atherosclerotic lesions. A recently identified secretion product of activated rodent macrophages is nitric oxide,[3] which contributes to host defence by its cytolytic and antimicrobial properties. It is synthesized from L-arginine by an inducible form of nitric oxide synthase (iNOS), with L-citrulline as a by-product. The expression of iNOS has been demonstrated in atherosclerotic vessels.[4]

The effect of foam cell transformation of J774 murine macrophages on the induction of iNOS activity has been reported.[5] It could be confirmed that this function is indeed inhibited in cells loaded with oxLDL but not with acetylated low density lipoproteins (acLDL). As this indicates that not lipid accumulation as such, but some constituent of oxLDL might exert an inhibitory action on the induction of iNOS activity, this study was designed to elucidate the underlying mechanism.

Since the induction of iNOS in macrophages is also inhibited by glucocorticoids (GC)[6] and glucocorticoids are synthesized from cholesterol via oxidative pathways, it was hypothesised that GC-like products might be formed during the oxidation of the cholesterol-rich LDL particle. To investigate whether a GC-like factor in oxLDL might be responsible for the observed inhibition of iNOS activity, the glucocorticoid receptor (GCR) antagonist mifepristone was added during lipid loading and stimulation, thus attempting to reverse the observed inhibition.

### Materials and Methods

*Materials:* All cell culture media and supplements were from Gibco Ltd, Paisley, UK. Reagents for the cholesterol assay were from Boehringer Mannheim (Mannheim, Germany), reagents for the protein assay from Pierce (Rockford, IL, USA), sodium nitrite from Merck (Darmstadt, Germany), tetramethoxypropane from Aldrich Chemie (Brussels, Belgium), and Aquacide I from Calbiochem (La Jolla, CA, USA). Interferon-γ (recombinant rat IFN-γ) was purchased from Holland Biotechnology (Leiden, The Netherlands). Mifepristone (RU 38 486) was a gift from Roussel UCLAF (Romainville, France). Lipoprotein deficient serum (LPDS), low density lipoprotein (LDL) and acetylated LDL (acLDL) were prepared as

© 1994 Rapid Communications of Oxford Ltd described previously.[7] All other reagents, lipopolysaccharide (LPS, *Salmonella typhosa*) and dexamethasone, were from Sigma Chemical Company (St. Louis, MO, USA).

*LDL oxidation:* LDL 4 mg/ml was dialysed for 24 h against phosphate buffered saline (PBS) to remove EDTA. Oxidation of LDL 200 μg/ml was performed in PBS by addition of $CuCl_2$ (6.4 μM) during 16 h at 37°C and stopped by adding EDTA (200 μM) and keeping on ice for 1 h. Before and after oxidation, a sample was taken for determination of thiobarbituric acid reactive substances (TBARS) as a measure of lipid peroxidation. Then the oxLDL solution was dialysed for 24 h against PBS plus EDTA to remove the $Cu^{2+}$ ions and concentrated to ±1.5 mg/ml by keeping the dialysis bag in Aquacide I powder for ± 8 h.

*Lipid peroxidation assay:* A sample volume of 0.5 ml was mixed with 2 ml 0.25 M HCl containing 3.75% thiobarbituric acid and 15% TCA, and boiled for 15 min. After centrifugation at 1500 **g** for 15 min, absorbance was read at 532 nm. Results, expressed as nmol malondialdehyde (MDA) equivalents/mg protein, are obtained from a standard curve with tetramethoxypropane, which decomposes to MDA under the assay conditions.

*Culture and lipid loading of J774 cells:* J774 murine macrophage-like cells (ATCC, Rockville, MD, USA) were maintained in Dulbecco's modified Eagle medium (DMEM) supplemented with 10% foetal calf serum (FCS) and antibiotics (penicillin 100 U/ml, streptomycin 100 μg/ml). Cells ($2 \times 10^6$/35 mm dish) were seeded in 6-well plates to obtain a confluent monolayer 24 h later. Subsequently, lipid loading was performed by incubation with oxLDL (200 μg/ml) or acLDL (100 μg/ml) for 24 h in DMEM supplemented with 10% lipoprotein deficient serum and antibiotics. The amount of total cell protein after loading with acLDL or oxLDL was 724 ± 84 μg and 777 ± 148 μg respectively, which is not different from control cells (690 ± 37 μg), indicating that lipid loading did not result in cell loss ($n = 4$). In some experiments polymyxin B (10 μg/ml) was added during loading.

*Determination of total cellular cholesterol and protein:* After lipid loading the monolayers were washed three times with cold PBS to remove serum proteins and lipoproteins. Cholesterol extraction was performed in the plate by adding 5 ml hexane:propan-2-ol (3:2) for 1 h, followed by a brief wash with 1 ml. The solvent was evaporated under reduced pressure and cholesterol and cholesterol esters determined by an enzymatic assay.[8] Total cell protein was determined by the bicinchoninic acid (BCA) method.[9] The wells were exposed to 3 ml trichloroacetic acid (TCA) 5% for 2 h, followed by dissolution of the precipitated proteins in 2 ml 0.1 N NaOH plus 0.5% sodium dodecyl sulphate. Results are expressed as μg cholesterol/mg protein and percent esters of total cholesterol.

*Stimulation of J774 cells:* Control and lipid-laden monolayers were washed three times with warm DMEM and fresh medium (DMEM without phenol red, 400 μM L-arginine, 10% FCS, antibiotics) was added. Cells were then stimulated with LPS 10 μg/ml with or without IFN-γ 100 U/ml for 48 h.

*Determination of nitric oxide synthase activity:* NOS activity was assessed by measuring nitrite, a stable NO metabolite, and L-citrulline in the cell-free supernatant 48 h after stimulation. The nitrite assay is based on the diazotization of sulfanilic acid by NO, derived from $NO_2^-$ at acidic pH. Subsequently, coupling with *N*-(1-naphthyl)-ethylenediamine yields a coloured product that is measured spectrophotometrically at 540 nm.[10] L-Citrulline was measured with a Biotronik LC 6000 E amino acid analyser (Biotronok, Maintal, Germany). Results are expressed as nmoles nitrite and citrulline/mg protein. The detection limit of both assays is 1 μM.

*Effect of dexamethasone and mifepristone on NOS activity:* Dexamethasone $10^{-5}$, $10^{-6}$ and $10^{-7}$ M was added to the control cells together with the stimulus. The ability of mifepristone, a glucocorticoid receptor (GCR) antagonist, to prevent the effects of dexamethasone, was investigated by co-addition ($10^{-5}$ and $10^{-7}$ M). Mifepristone $10^{-5}$ and $10^{-7}$ was also added during lipid loading and stimulation of foam cells to assess its ability to prevent the inhibitory effect of oxLDL.

*Statistical analysis:* Values are expressed as the mean ± S.E.M. of four to five experiments. Differences between the means were determined by the Student's paired *t*-test. A *p*-value less than 0.05 was considered significant.

### Results

*LDL oxidation:* LDL contained very low amounts of MDA equivalents before oxidation, indicating that minimal spontaneous oxidation had occurred. Oxidation with $CuCl_2$ increased the MDA equivalents 42-fold, while final dialysing and concentrating resulted in substantial loss of TBARS (Table 1).

**Table 1:** LDL oxidation, measured as thiobarbituric acid reactive substances (TBARS)

| LDL | TBARS (nmol MDA equivalents/mg protein) |
|---|---|
| Before oxidation | 1.5 ± 0.8 ($n = 8$) |
| After oxidation | 64.0 ± 4.0 ($n = 8$) |
| After dialysis | 6.9 ± 0.7 ($n = 6$) |

*Foam cell formation:* The total cholesterol content of control macrophages was 30 ± 2 μg/mg protein, 94 ± 2% was present as free cholesterol. Incubation of J774 cells with acLDL 100 μg/ml or oxLDL 200 μg/ml resulted in foam cells with a comparable high amount of total cholesterol, 101 ± 10 and 92 ± 17 μg cholesterol/mg protein respectively. The percentage of cholesterol esters was 53% and 36% respectively.

*Nitrite and citrulline release by unloaded J774 cells:* Supernatant of unstimulated cells did not contain measurable amounts of nitrite or citrulline (Fig. 1). LPS 10 μg/ml induced NOS activity, as indicated by the accumulation of 59 ± 15 nmoles nitrite and 98 ± 15 nmoles citrulline/mg protein in the 48 h supernatant. Addition of IFN-γ resulted in significantly higher production of these two L-arginine metabolites, especially citrulline. Interferon-γ alone was only a very weak stimulus for NOS induction (data not shown).

*Nitrite and citrulline release by J774 foam cells:* Unstimulated acLDL-laden foam cells did not produce nitrite or citrulline (Fig. 1). However, in oxLDL-laden cells NOS activity was induced, resulting in small amounts of nitrite (13 ± 4 nmol/mg protein) and citrulline (12 ± 5 nmol/mg protein) in the 48 h supernatant of unstimulated cells.

LPS 10 μg/ml induced NOS activity in acLDL-laden cells to the same extent as in control cells. In oxLDL-laden cells however, NOS activity was significantly suppressed, with only 38% of nitrite production and 25% of citrulline production compared to control cells. This was also observed for the stronger stimulation with both LPS and IFN-γ, although the inhibition was less pronounced (75% of nitrite release and 33% of citrulline release compared to control cells).

Addition of polymyxin B together with oxLDL during lipid loading did not eliminate the small induction of NOS activity nor the suppression of LPS induced activity in these cells (data not shown).

*Effect of dexamethasone and mifepristone on NOS activity in J774 cells:* In control cells, addition of dexamethasone together with LPS 10 μg/ml resulted in dose dependent inhibition of nitrite release (Table 2). The inhibitory effect of dexamethasone was reversed in a concentration dependent manner by co-addition of the glucocorticoid receptor antagonist mifepristone (Table 2, Fig. 2). Mifepristone did not affect nitrite production when added alone (data not shown).

Mifepristone ($10^{-7}$ M and $10^{-5}$ M), added during lipid loading and stimulation of the cells, failed to antagonize the inhibition of NOS activity in the oxLDL-laden foam cells (Fig. 2).

**Table 2:** Effect of dexamethasone and mifepristone on LPS induced nitrite release

| Concentration of dexamethasone (M) | Control | Concentration of mifepristone | |
|---|---|---|---|
| | | $10^{-7}$ M | $10^{-5}$ M |
| $10^{-7}$ | 66 ± 3 | 77 ± 2* | 89 ± 4* |
| $10^{-6}$ | 34 ± 3 | 74 ± 3* | 89 ± 4* |
| $10^{-5}$ | 20 ± 2 | 52 ± 3* | 77 ± 2* |

Results are expressed as percent of maximal LPS induced nitrite production ± S.E.M. ($n = 4$).
*$p < 0.05$, different from dexamethasone alone.

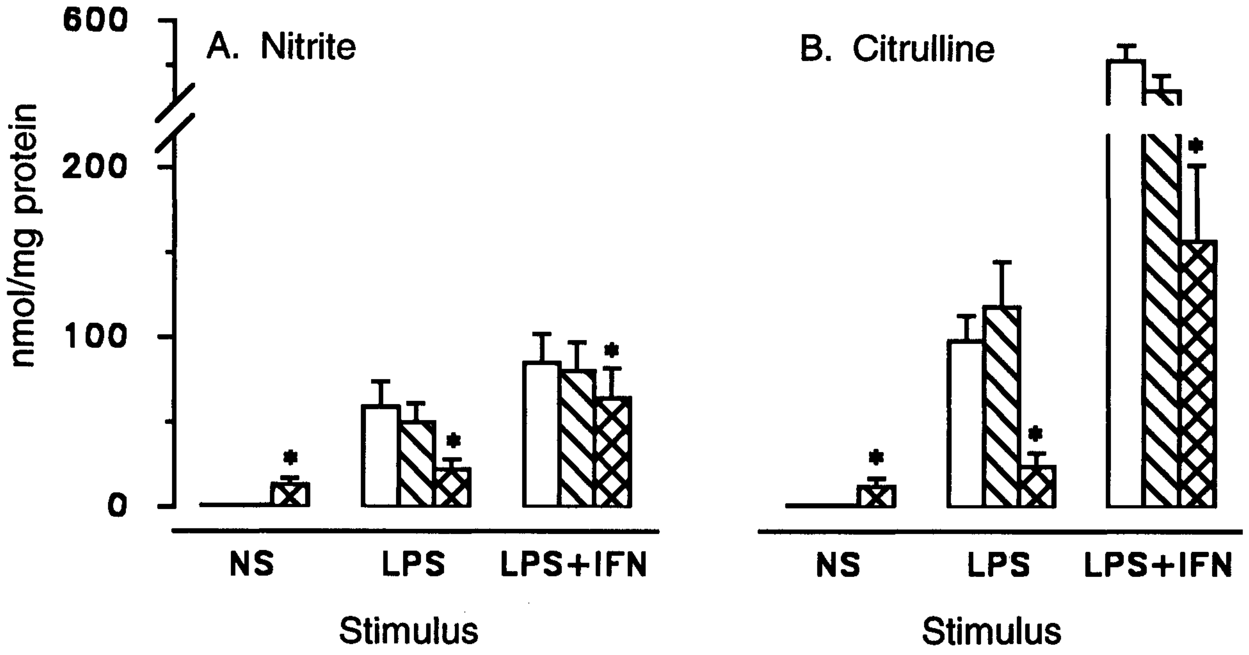

FIG. 1. Nitrite (A) and citrulline (B) release by J774 control and foam cells. Nitrite and citrulline were measured in the supernatant of control cells (□), acLDL-laden cells (▨) and oxLDL-laden cells (▩) after 48 h with or without LPS ± IFN-γ. NS = not stimulated. *$p < 0.05$ *vs.* control.

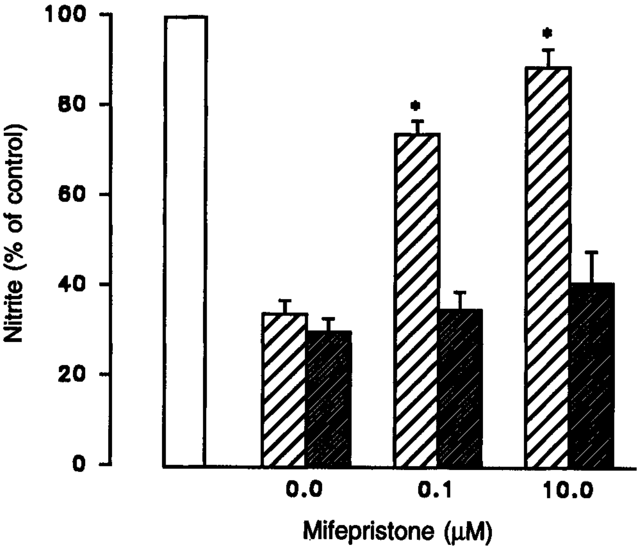

FIG. 2. Mifepristone reverses induced NOS inhibition by dexamethasone ($10^{-6}$ M) (▨) without influencing the suppression by oxLDL (■). Control cells (□) were stimulated with LPS (10 μg/ml) and produced 59 ± 15 nmol nitrite/mg protein. Results are expressed as percent of control. $^{*}p < 0.05$ *vs.* no mifepristone added.

### Discussion

J774 cells have been used for the study of cholesterol metabolism and lipoprotein handling[7,11] in macrophages. They express the scavenger receptor(s) and thus can take up modified lipoproteins such as acLDL or oxLDL, eventually leading to intracellular cholesterol ester accumulation, the hallmark of foam cells. Several authors have reported the presence of oxLDL in atherosclerotic lesions,[12,13] whereas acLDL is probably not relevant *in vivo*. *In vitro*, both forms of modified lipoproteins induce lipid accumulation in J774 cells, and thus provide an easy-to-use foam cell model. In the present experiments, oxLDL had to be used in a higher concentration than acLDL in order to obtain cells with comparable amounts of total cholesterol after 24 h. Also, cholesterol esterification is higher in acLDL-laden cells. These two observations point to differences in uptake and intracellular processing of the modified lipoproteins. As total cell protein after lipid loading was not affected, this procedure proved to be non-toxic to the macrophages.

In this study NOS activity in J774 cells was induced by LPS. NOS metabolizes L-arginine to NO and citrulline, which both accumulate extracellularly. Nitric oxide spontaneously converts to the more stable products nitrite and nitrate, about 60% and 40% respectively, with no conversion from one product to the other.[14] Nitric oxide is presumably the main source of nitrite, whereas citrulline is involved in more complex biochemical pathways and can also be derived from L-arginine through the consecutive action of arginase and ornithine transcarbamylase. IFN-γ, which on its own was a very weak stimulus for NOS induction in J774 cells,[3] acted synergistically with LPS to induce higher NOS activity, as indicated by the increased release of nitrite and citrulline. This combination possibly also stimulated the arginase pathway, since the synergistic effect of IFN-γ for citrulline release was much higher than for nitrite release. On the other hand, LPS plus IFN-γ may have stimulated the macrophages for production of oxygen radicals, and reaction of superoxide anion and NO preferentially leads, via peroxynitrite, to nitrate,[15] at the expense of nitrite. In that case, citrulline is a better parameter of NOS activity than nitrite. Another explanation could be leakage of intracellular L-citrulline[16] in the supernatant, due to cell death. In view of these considerations, determination of both nitrite and citrulline in the cell-free supernatant seems to be required to evaluate induced NOS activity.

NOS activity was investigated in J774 foam cells. AcLDL-laden cells, unstimulated or stimulated, were not different from control cells with respect to either nitrite or citrulline release, indicating that lipid accumulation as such did not necessarily alter induced NOS activity. However, in oxLDL-laden cells, two changes were observed. First, unstimulated oxLDL-laden cells already displayed some induced NOS activity, whereas acLDL-laden cells or control cells did not. This small induction of NOS activity was apparently not the result of LPS contamination in the oxLDL preparation since addition of the LPS-binding antibiotic polymyxin B did not prevent it. Secondly, the subsequent stimulation of oxLDL-laden cells with LPS or LPS plus IFN-γ resulted in lower amounts of released nitrite, compared to acLDL-laden cells or control cells. This is not due to trapping of NO or preferential conversion of NO to nitrate by the oxLDL, since citrulline decreased as well. It is not clear from the present experiments whether the induction of the NOS gene, the activity of the NOS enzyme or the availability of L-arginine is decreased. It has been reported that oxLDL inhibits other LPS inducible gene products such as TNFα,[17] IL-1α and β,[18] IL-6[18] and PDGF.[19,20] In these studies, mRNAs were suppressed, suggesting that inhibition took place at the level of transcription or signal transduction. The observation that oxLDL inhibits the induction of several cytokines and NOS points to a more general mechanism of action. Since glucocorticoids also inhibit the induction of many LPS inducible genes,[21] it was speculated that a common mechanism might be responsible.

It could be that cholesterol oxidation products with glucocorticoid-like activity are formed during the oxidation of LDL. However, although the GC receptor antagonist mifepristone was clearly capable of partially reversing dexamethasone mediated NOS inhibition, it was unable to prevent the inhibitory action of oxLDL on NOS activity.

Other cellular events initiated by oxLDL may be involved. For instance, it has been reported that oxLDL increases arachidonate metabolism to prostaglandin $E_2$ ($PGE_2$) and $PGI_2$,[22] and some of these products may inhibit NOS induction.[23]

TNFα has been reported to be an autocrine mediator in NOS induction in murine macrophages.[24,25] In oxLDL-treated cells, TNFα expression after stimulation is decreased,[17] which could lead to decreased NOS induction. However, oxLDL did not induce TNFα transcription[17] in unstimulated murine cells, whereas NOS induction was observed. Although TNFα expression and NO production can occur independently, the possibility that the oxLDL-mediated NOS inhibition is TNF-dependent remains open.

In conclusion, the study demonstrated that induction of NOS activity is specifically inhibited in oxLDL- and not in acLDL-laden foam cells. The mechanism of the observed inhibition is not clear at this moment, but a glucocorticoid-like action of oxLDL does not seem to be involved in view of the failure of the GCR antagonist to prevent the suppression.

### References

1. Gerrity RG. The role of the monocyte in atherogenesis. I. Transition of blood-bone monocytes into foam cell in fatty lesions. *Am J Pathol* 1981; **103:** 181–190.
2. Witztum JL. Role of oxidised low density lipoprotein in atherogenesis. *Br Heart J* 1993; **69(Suppl.):** S12–S18.
3. Stuehr DJ, Marletta MA. Synthesis of nitrite and nitrate in murine macrophage cell lines. *Cancer Res* 1987; **47:** 5590–5594.
4. Verbeuren TJ, Bonhomme E, Laubie M, Simonet S. Evidence for induction of non-endothelial nitric oxide synthase in aortas of cholesterol-fed rabbits. *J Cardiovasc Pharmacol* 1993; **21:** 841–845.
5. Jorens PG, Rosseneu M, Devreese AM, Bult H, Marescau B, Herman AG. Diminished capacity to release metabolites of nitric oxide synthase in macrophages loaded with oxidized low-density lipoproteins. *Eur J Pharmacol* 1992; **212:** 113–115.
6. Di Rosa M, Radomski M, Carnuccio R, Moncada S. Glucocorticoids inhibit the induction of nitric oxide synthase in macrophages. *Biochem Biophys Res Commun* 1990; **172:** 1246–1252.
7. Vercaemst R, Union A, Rosseneu M. Separation and quantitation of free cholesterol and cholesteryl esters in a macrophage cell line by high performance liquid chromatography. *J Chromatogr* 1989; **494:** 43–52.
8. Salè FO, Marchesini S, Fishman PH, Berra B. A sensitive enzymatic assay for determination of cholesterol in lipid extracts. *Anal Biochem* 1984; **142:** 347–350.
9. Smith PK, Krohn RI, Hermanson GT, *et al.* Measurement of protein using bicinchoninic acid. *Anal Biochem* 1985; **150:** 76–85.
10. Ignarro LJ, Buga GM, Wood KS, Byrns RE, Chaudhuri G. Endothelium-derived relaxing factor produced and released from artery and vein in nitric oxide. *Proc Natl Acad Sci USA* 1987; **84:** 9265–9269.
11. Roma P, Catapano AL, Bertulli SM, Varesi L, Fumagalli R, Bernini F. Oxidized LDL increase free cholesterol and fail to stimulate cholesterol esterification in murine macrophages. *Biochem Biophys Res Commun* 1990; **171:** 123–131.
12. Ylä-Herttualla S, Palinski W, Rosenfeld ME, *et al.* Evidence for the presence of oxidatively modified low density lipoprotein in atherosclerotic lesions of rabbit and man. *J Clin Invest* 1989; **84:** 1086–1095.
13. Haberland ME, Fong D, Cheng L. Malondialdehyde-altered protein occurs in atheroma of Watanabe heritable hyperlipidemic rabbits. *Science* 1988; **241:** 215–218.
14. Stuehr DJ, Marletta MA. Synthesis of nitrite and nitrate in murine macrophage cell lines. *Cancer Res* 1987; **47:** 5590–5594.
15. Murphy ME, Sies H. Reversible conversion of nitroxyl anion to nitric oxide by superoxide dismutase. *Proc Natl Acad Sci USA* 1991; **88:** 10860–10864.
16. Baydoun AR, Bogle RG, Pearson JD, Mann GE. Selective inhibition by dexamethasone of induction of NO synthase, but not of induction of L-arginine transport, in activated murine macrophage J774 cells. *Br J Pharmacol* 1993; **110:** 1401–1406.
17. Hamilton TA, Ma G, Chisolm GM. Oxidized low density lipoprotein suppresses the expression of tumor necrosis factor-α mRNA in stimulated murine peritoneal macrophages. *J Immunol* 1990; **144:** 2343–2350.
18. Fong LG, Fong TA, Cooper AD. Inhibition of lipopolysaccharide-induced interleukin-1 beta mRNA expression in mouse macrophages by oxidized low density lipoprotein. *J Lipid Res* 1991; **32:** 1899–1910.
19. Malden LT, Chait A, Raines EW, Ross R. The influence of oxidatively modified low density lipoproteins on expression of platelet-derived growth factor by human monocyte-derived macrophages. *J Biol Chem* 1991; **266:** 13901–13907.
20. Fox PL, Chisolm GM, DiCorleto PE. Lipoprotein-mediated inhibition of endothelial cell production of platelet-derived growth factor-like protein depends on free radical lipid peroxidation. *J Biol Chem* 1987; **262:** 6046–6054.
21. Fantuzzi G, Ghezzi P. Glucocorticoids as cytokine inhibitors: role in neuroendocrine control and therapy of inflammatory diseases. *Mediators of Inflammation* 1993; **2:** 263–270.
22. Yokode M, Kita T, Kikawa Y, Ogorochi T, Narumiya S, Kawai C. Stimulated arachidonate metabolism during foam cell transformation of mouse peritoneal macrophages with oxidized low density lipoprotein. *J Clin Invest* 1988; **81:** 720–729.
23. Marotta P, Santebin L, Di Rosa M. Modulation of the induction of nitric oxide synthase by eicosanoids in the murine macrophage cell line J774. *Br J Pharmacol* 1992; **107:** 640–641.
24. Draper JC, Wietzerbin J, Hibbs JB. Interferon-γ and tumor necrosis factor induce the L-arginine-dependent cytotoxic effector mechanism in murine macrophages. *Eur J Immunol* 1988; **18:** 1587–1592.
25. Langermans JAM, Van Der Hulst MEB, Nibbering PH, Hiemstra PKS, Fransen L, Van Furth R. IFN-γ-induced L-arginine-dependent toxoplasmastatic activity in murine peritoneal macrophages is mediated by endogenous tumor necrosis factor. *J Immunol* 1992; **148:** 568–574.

ACKNOWLEDGEMENTS: The work was supported by The Belgian Programme on Interuniversity Poles of Attraction Initiated by the Belgian State, Prime Minister's Office, Science Policy Programming and by the Belgian Fund for Medical Research.

**Received 6 April 1994; accepted 28 April 1994**
